# The complete mitochondrial genome of *Alternaria alternata* (Hypocreales: Nectriaceae)

**DOI:** 10.1080/23802359.2017.1372720

**Published:** 2017-09-01

**Authors:** Min Liao, Cheng Chen, Qiang Li

**Affiliations:** a College of Life Science & Biotechnology, Mianyang Normal University, Mianyang, China;; b Institute of Plant Protection, Sichuan Academy of Agricultural Sciences, Chengdu, China;; c Biotechnology and Nuclear Technology Research Institute, Sichuan Academy of Agricultural Sciences, Chengdu, China

**Keywords:** *Alternaria alternata*, mitogenome, phylogenetic analysis

## Abstract

In the present study, we presented the complete mitochondrial genome of *Alternaria alternata*. It has a total length of 50, 107 bp, the base composition of this mitogenome is as follows: A (35.9%), T (34.9%), C (14.4%), and G (14.8%). The mitogenome contains 26 protein-coding genes, two ribosomal RNA (rRNA) genes, and 25 transfer RNA (tRNA) genes. The taxonomic status of the *A. alternata* mitogenome exhibits a closest relationship with *Phaeosphaeria nodorum*.

The genus *Alternaria* is a group of infectious pathogenic fungi that not only invade a wide range of crops but also induce severe allergic reactions (Wiest et al. 1987; Singh et al. 2016), such as hypersensitivity pneumonitis, asthma and allergic fungal rhinitis and sinusitis, in a part of the human population (Kustrzeba-Wójcicka et al. 2014). There are 299 species in the genus, they are ubiquitous in the environment and are a natural part of fungal flora almost everywhere (Woudenberg et al. 2015). This ascomycete is pathogenic on various plant hosts and produces host-selective toxins (Tsuge et al. 2013; Armitage et al. 2015). At least 20% of agricultural spoilage is caused by *Alternaria* species, and most severe losses may reach up to 80% of yield. To the best of our knowledge, this is the first report on the complete mitochondrial DNA of *A. alternate*, which will provide a reference for understanding the phylogeny and evolution of the genus *Alternaria*.

The specimen (*A. alternata*) was isolated from the vegetables-growing soil in Mianyang, Sichuan, China (104.73E; 31.48 N) and was stored in Sichuan Academy of Agricultural Sciences (No. TKL50). The total genomic DNA of *A. alternata* was extracted using Fungal DNA Kit D3390-00 (Omega Bio-Tek, Norcross, GA) and purified through a Gel Extraction Kit (Omega Bio-Tek, Norcross, GA). Purified DNA was stored in the sequencing company (BGI Tech, Shenzhen, China). Sequencing libraries were constructed with purified DNA following the instructions of NEBNext^®^ Ultra™ II DNA Library Prep Kit (NEB, Beijing, China). Whole genomic sequencing was performed by the Illumina HiSeq 2500 Platform (Illumina, San Diego, CA). Multiple steps were used for quality control and *de novo* assembly of the mitogenome according to Bi (2017). The obtained clean reads were screened out by bowtie2 (Langmead and Salzberg 2012) using other mitochondrial genomes of closely related species as references, and then assembly as implemented by SPAdes 3.9.0 (Bankevich et al. 2012). Gaps among contigs were filled by using MITObim V1.9 (Hahn et al. 2013). The determined genome was annotated using the MFannot tool (http://megasun.bch.umontreal.ca/cgi-bin/mfannot/mfannotInterface.pl), combined with manual corrections. tRNAs were annotated by ARWEN Web Server (Laslett and Canbäck 2008).

The total length of *A. alternata* circular mitogenome is 50, 107 bp. This mitogenome was submitted to GenBank database under accession no. MF669499. The circular mitogenome contains 26 protein-coding genes, two ribosomal RNA genes (rrnS and rrnL), and 25 transfer RNA (tRNA) genes. The base composition of the genome is as follows: A (35.9%), T (34.9%), C (14.4%), and G (14.8%).

To validate the phylogenetic position of *A. alternata*, the genome-wide alignment of *A. alternata* mitogenomes and eight closely related species was constructed by HomBlocks (https://github.com/fenghen360/HomBlocks) (Bi 2017; Bi et al. 2017). Bayesian analysis (BI) and maximum likelihood (ML) were used to construct the phylogenetic trees with all protein-coding genes and rRNA according to Bi (2017). Bootstrap values were calculated using 1000 replicates to assess node support. As shown in the phylogenetic tree (Figure 1), the taxonomic status of the *A. alternata* based on mitogenome exhibits a closest relationship with *P. nodorum* (GenBank accession number: EU053989).

**Figure 1.**
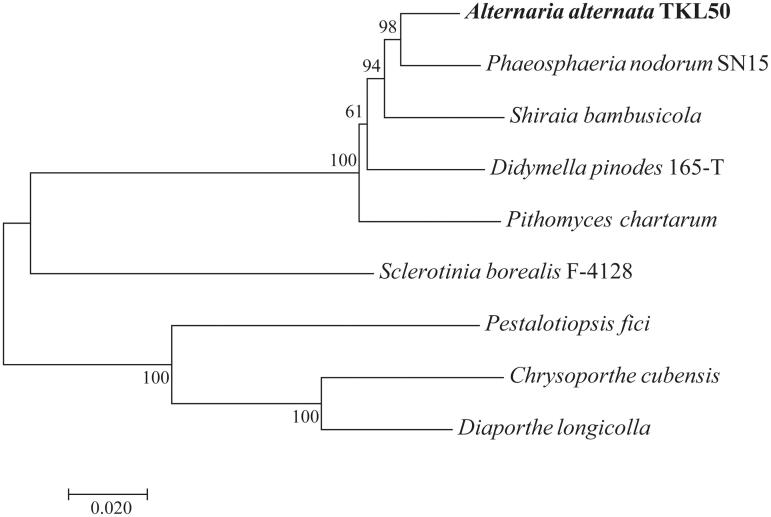
Phylogenetic relationships among nine fungal mt genomes. This tree was drawn without setting of a outgroup. The length of branch represents the divergence distance. Mitogenome accession numbers used in this phylogeny analysis: *P. nodorum* (EU053989), *S. bambusicola* (KM382246), *D. pinodes* (KT946597), *P. chartarum* (KY792993), *S. borealis* (KJ434027), *P. fici* (KX870077), *C. cubensis* (KT380885), and *D. longicolla* (KP137411).
